# Somatization, psychological distress, and quality of life across fibromyalgia, irritable bowel syndrome, and their comorbid phenotype: a cross-sectional clinical comparison

**DOI:** 10.3389/fpsyg.2025.1709017

**Published:** 2025-12-04

**Authors:** Laura Prospero, Giuseppe Riezzo, Gaetana Laselva, Benedetta D’Attoma, Antonia Ignazzi, Francesco Goscilo, Antonella Bianco, Isabella Franco, Nicola Verrelli, Claudia Beatrice Bagnato, Sergio Coletta, Maria Grazia Refolo, Francesco Russo

**Affiliations:** 1 Functional Gastrointestinal Disorders Research Group, National Institute of Gastroenterology “Saverio de Bellis”, IRCCS Hospital, Castellana Grotte, Italy; 2 Rheumatology Outpatient Clinic, National Institute of Gastroenterology "Saverio de Bellis", IRCCS Hospital, Castellana Grotte, Italy; 3 Laboratory of Movement and Wellness, National Institute of Gastroenterology "Saverio de Bellis", IRCCS Hospital, Castellana Grotte, Italy; 4 Core Facility Biobank, National Institute of Gastroenterology "Saverio de Bellis", IRCCS Hospital, Castellana Grotte, Italy; 5 Laboratory of Clinical Pathology, National Institute of Gastroenterology "Saverio de Bellis", IRCCS Hospital, Castellana Grotte, Italy

**Keywords:** fibromyalgia, irritable bowel syndrome, gastrointestinal symptoms, gut-brain axis, psychological profile, quality of life

## Abstract

**Background:**

Fibromyalgia (FM) and irritable bowel syndrome (IBS) are increasingly recognized as disorders involving central sensory processing and gut–brain axis dysregulation, often accompanied by autonomic and psychological disturbances.

**Methods:**

We investigated whether patients with comorbid FM and IBS (FM + IBS) experience greater somatization and reduced quality of life (QoL) compared to those with either condition alone, and if somatization serves as a predictor of gastrointestinal (GI) symptom severity. In this cross-sectional study, 53 adults (mean age 47.4 ± 1.3 years; 48 women) were classified into three groups: FM-only (*n* = 13), IBS-only (*n* = 18), and FM + IBS (*n* = 22). Participants completed validated assessments including the IBS Symptom Severity Scale, the Symptom Checklist-90-Revised, the Perceived Stress Scale, and QoL measures (SF-36, WHOQOL-BREF). Group differences were analyzed using the Kruskal-Wallis test, and predictors of IBS severity were identified via stepwise multiple linear regression.

**Results:**

FM-only and FM + IBS patients reported similar levels of pain, fatigue, and functional impact. GI symptoms were mild in FM-only patients but moderate in IBS-only and FM + IBS groups, with large effect sizes for psychological distress, mental health, and IBS severity. While FM + IBS participants showed slightly higher FM-related symptom scores, differences were not statistically significant. Somatization and diagnostic group independently predicted IBS severity, together explaining 50% of the variance.

**Conclusion:**

These findings demonstrate a progressive increase in somatization and a parallel decline in QoL across the spectrum from IBS-only to FM-only to FM + IBS, supporting the concept of functional somatic syndromes as a continuum. Incorporating routine assessment of somatization and QoL impairment may help identify patients at higher risk of treatment resistance and facilitate timely, integrated biopsychosocial strategies, including cognitive-behavioral and neuromodulatory interventions.

## Introduction

Fibromyalgia (FM) is a chronic, female-predominant functional somatic syndrome affecting about 2 to 4% of the global population, with gender differences likely due to hormonal, psychosocial, and diagnostic factors (Bair and Krebs, 2020; Castro-Sanchez et al., 2012). FM is marked by widespread musculoskeletal pain, fatigue, cognitive difficulties (“fibro-fog”), and gastrointestinal (GI) symptoms. Importantly, 30–50% of patients experience GI disturbances severe enough to need clinical evaluation (Chinn et al., 2016). Psychological comorbidities are common, with mood disorders reported in 13–80% of patients and major depressive disorder in 19–65% (Galvez-Sanchez et al., 2019; Kleykamp et al., 2021; Pacho-Hernandez et al., 2024), which increases disease burden and fuels the misconception of FM as a solely psychosomatic condition.

Diagnosis is often delayed by two to 5 years from symptom onset (Wolfe et al., 2011), reflecting challenges in clinical recognition. While chronic pain is its hallmark feature (Wolfe et al., 2016), the underlying etiology remains unclear (de Tommaso et al., 2022). Proposed mechanisms include genetic predisposition (Lukkahatai et al., 2018), central sensitization (Rehm et al., 2021), neuroinflammation (Sarzi-Puttini et al., 2020), and alterations in gut microbiota (Minerbi et al., 2023). FM frequently overlaps with disorders of gut–brain interaction (DGBIs), with 20–40% of patients meeting criteria for a DGBI (Sperber et al., 2021).

Among DGBIs, irritable bowel syndrome (IBS) is the most common comorbidity, affecting 3–10% of the global population, with a similar 2:1 female predominance (Sperber et al., 2021; Yunus, 2001). IBS prevalence varies across regions (10–15%) (Mearin et al., 2016), but its clinical presentation consistently reflects gut–brain axis dysregulation, and is characterized by recurrent abdominal pain, bloating, and altered bowel habits (Chey et al., 2015). Its diagnosis is based on Rome IV criteria, which emphasize standardized symptom thresholds and the exclusion of organic disease (Drossman and Hasler, 2016).

Evidence indicates a substantial comorbidity between FM and IBS. IBS patients face an 80% increased risk of developing FM (Cole et al., 2006), and longitudinal studies reveal a bidirectional relationship, with each condition doubling the risk of the other over 5 years (Erdrich and Harnett, 2025). Psychological disturbances prevalent in FM, particularly depression and anxiety (Eik-Nes et al., 2022), are strongly associated with IBS and functional dyspepsia (Erdrich and Harnett, 2025). Conversely, IBS-like GI symptoms are disproportionately common in FM populations and correlate with FM severity (Enkvist et al., 2023; Valencia et al., 2022). This bidirectionality suggests that FM severity extends beyond pain and fatigue to include emotional distress, maladaptive personality traits, such as neuroticism and catastrophizing (Doreste et al., 2024) and psychosomatic processes.

Patients with both FM and IBS exhibit poorer psychological well-being and reduced quality of life (QoL) compared to those with either condition alone (Sperber et al., 1999), and multivariate analyses show higher healthcare utilization (Tarar et al., 2023). Neurobiological models propose that central sensitization links these conditions, amplifying neural signaling within a hyper-excitable pain network (Adams and Turk, 2015). This framework emphasizes the importance of assessing psychopathological profiles, personality traits, and somatization to design integrated treatment strategies and reduce diagnostic delays (Taylor et al., 2019).

Despite guidelines advocating for multidisciplinary care in FM, fewer than 20% of patients receive coordinated GI and rheumatologic care (Jones et al., 2024). Moreover, although clinical experience suggests a substantial overlap in symptom burden across FM and IBS, there is still a lack of systematic data comparing GI and psychological features, such as anxiety, depression, and somatization, among FM-only, IBS-only, and FM + IBS groups (Prospero et al., 2021).

This cross-sectional observational study aimed to identify distinct somatic and psychological profiles in patients with FM + IBS compared to FM-only and IBS-only groups, and evaluate differences in QoL using validated instruments. We hypothesized that comorbid FM + IBS would represent a high-burden phenotype characterized by additive impairment in somatization and QoL, and that somatization would independently predict GI symptom severity.

## Materials and methods

### Subject recruitment

**Figure 1:**
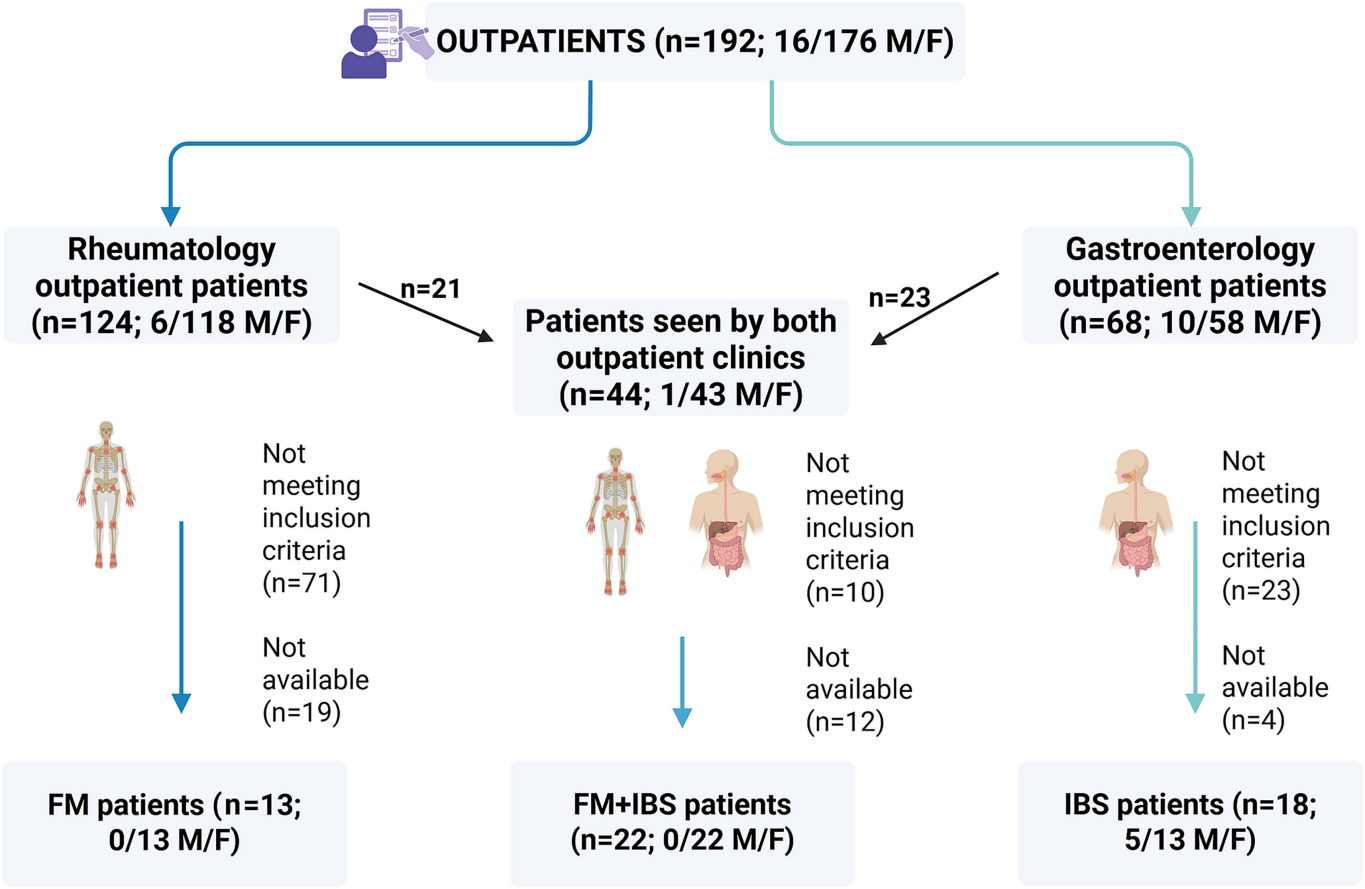
The flowchart of the study.

Between September 2023 and March 2025, 192 outpatients (16 males, 176 females; aged 18–65 years) were contacted from two clinics at the National Institute of Gastroenterology “Saverio de Bellis,” IRCCS Hospital, Italy (Figure 1). From the Rheumatology Outpatient Clinic, 124 patients (6 males, 118 females) were assessed. After excluding 71 who did not meet the inclusion criteria and 19 who declined, 13 female patients with FM were identified.

From the Functional Gastrointestinal Disorders Research Unit, 68 patients (10 males, 58 females) were evaluated. Four declined participations, and 23 did not meet the inclusion criteria, resulting in 18 patients diagnosed with IBS (5 males, 13 females). Additionally, 44 patients (1 male and 43 females) from both clinics were assessed for comorbidities. Of these, 12 were unavailable, and 10 did not meet the diagnostic criteria, leading to the identification of 22 female patients with both FM and IBS (FM + IBS).

Patients with documented FM and/or IBS, confirmed through clinical evaluation and standard diagnostics, were included. Recent baseline blood tests, gastroscopy, and colonoscopy reports were required, along with tissue transglutaminase and anti-endomysium antibody tests to rule out celiac disease. No restrictive diets were necessary before inclusion.

Exclusion criteria included pregnancy; infectious, inflammatory, autoimmune, or neoplastic diseases; metabolic, endocrine, liver, kidney, or cardiovascular disorders; history of abdominal surgery; use of antibiotics or probiotics within the past 3 months; participation in high-intensity exercise (>5 h/week); and DSM-5 diagnoses of major psychiatric disorders (e.g., Somatic Symptom Disorder, autism spectrum disorders, schizophrenia, or severe mood or personality disorders). All participants provided written informed consent, and reasons for discontinuation were documented.

The study was conducted as part of a larger research project approved by the local Scientific Committee and the Institutional Ethics Committee of the IRCCS Ospedale Oncologico di Bari “Istituto Oncologico Giovanni Paolo II” (Protocol No. 117, approved March 3, 2023). It is registered on ClinicalTrials.gov (identifier: NCT06166563).

### Diagnosis of fibromyalgia

FM was diagnosed using the 2016 American College of Rheumatology (ACR) criteria: widespread pain index (WPI) ≥ 7 and symptom severity (SS) scale ≥ 5, or WPI 4–6 and SS ≥ 9, with symptoms present for at least 3 months, and no alternative disorder explaining the pain. Assessments included:

- Fibromyalgia Impact Questionnaire—Revised (FIQ-R): This tool consists of 21 items; each rated on a 0–10 scale grouped into physical function, overall impact, and symptoms (higher scores = greater impact) (Lee, 2021);
- Fatigue Severity Scale (FSS): this scale evaluates the severity of fatigue using a 7-point Likert scale across 9 items rated 1–7 (higher scores = greater fatigue) (Krumina et al., 2014);
- Digital Tender Point Examination: evaluates tenderness in response to manual pressure applied to 18 specific anatomical sites (Cott et al., 1992);
- Visual Analogue Scale (VAS) for pain: assesses patient-perceived pain intensity using a 10 cm horizontal line, ranging from 0 (no pain) to 100 mm (worst pain imaginable) (Crawford et al., 2011).

### Identification of patients with IBS

During outpatient evaluation, patients were diagnosed with IBS based on their medical history and the Rome IV criteria. To meet these criteria, patients reported recurrent abdominal pain occurring at least 1 day per week over the past 3 months, along with at least two of the following: pain related to defecation, changes in stool frequency, or changes in stool form (Lacy and Patel, 2017).

The symptom assessment was performed using the IBS Symptom Severity Score (IBS-SSS) (Francis et al., 1997). The IBS-SSS is a validated instrument designed to quantify symptom severity in individuals with IBS. It includes five items: “Abdominal pain intensity,” “Abdominal pain frequency,” “Abdominal bloating severity,” “Dissatisfaction with bowel habits,” and “Symptom impact on daily life.” The total score ranges from 0 to 500, and symptom severity is categorized as follows: mild = 75–175, moderate = 175–300, and severe > 300.

### Psychological questionnaires

#### Perceived stress scale (PSS)

The PSS provides a reliable method for assessing how stressful people perceive their daily lives. It reflects a person’s subjective experience of stress over the past month. This period strikes a balance, long enough to avoid daily fluctuations but short enough to show current stress levels. Scores range from 0 to 40, with higher scores indicating more perceived stress (Cohen et al., 1983).

#### Symptom checklist-90- revised (SCL-90-R)

This comprehensive tool evaluates psychological distress across nine key areas, from depression to psychoticism. While it encompasses multiple dimensions, we focused on the Global Severity Index (GSI) as it most effectively measures overall distress levels. After converting raw scores to T-scores, we applied the clinical cutoff of 63 to identify significant psychological symptoms (Jensen et al., 2013).

### Quality of life (QoL) questionnaires

#### 36-item short-form health survey (SF-36)

The SF-36 is a brief questionnaire used to assess QoL and overall health status, regardless of specific disease. It comprises multiple subscales categorized as follows:

Physical well-being: This is measured by subscales addressing physical activity, role limitations due to physical issues, and physical pain.
Intermediate subscales: These assess vitality and overall health.
Psychological health: The last three subscales focus on social activity limitations and role restrictions caused by emotional or mental health issues.

Scores from these subscales are summed, coded, and converted into a 0–100 scale, where 0 represents the worst possible health state and 100 the best. These scores can also be combined into two summary components: the Mental Component Summary (MCS) for mental health and the Physical Component Summary (PCS) for physical health (Barile et al., 2016).

#### World Health Organization quality of life-BREF (WHOQOL-BREF)

The WHOQOL-BREF is an abbreviated version of the WHOQOL-100 questionnaire developed by the World Health Organization to assess QoL from a multidimensional and cross-cultural perspective. It is a self-assessment tool consisting of 26 questions that investigate the individual’s perception of their QoL over the past 2 weeks. The questionnaire assesses QoL across four main domains: physical health, psychological health, social relations, and environment. Each question presents five response options on a Likert scale. The raw scores are then transformed to obtain a scaled score, expressed on a range from 0 to 100. Higher scores indicate a better perceived QoL (The WHOQOL Group, 1998).

### Laboratory tests

Blood samples were collected between 8:00 and 9:00 a.m. after an overnight fast. Complete blood count was performed using the Sysmex XT-1000 hematology analyzer (Dasit, Cornaredo, Milan, Italy). Biochemical parameters, including fasting glucose, insulin, electrolytes, creatinine, urea, lipid profile, liver enzymes, vitamin D, parathyroid hormone, high-sensitivity C-reactive protein, lactate dehydrogenase, creatine kinase, and thyroid profile, were measured using the COBAS 8000 autoanalyzer (Roche Diagnostics, Monza, Italy).

### Statistical analysis

The primary outcome was to assess baseline differences in GI symptom severity across three patient groups: FM-only, IBS-only, and FM + IBS. Secondary outcomes included comparisons of psychological distress, QoL, and functional impact.

Continuous variables are presented as mean ± standard error of the mean (SEM). Group comparisons were performed using the Kruskal–Wallis test, followed by Dunn’s post-hoc test for multiple comparisons. Categorical variables were analyzed using the *χ*^2^ test. Spearman’s rank correlation coefficient was used to assess bivariate associations.

A multivariable linear regression model was fitted to identify independent predictors of IBS-SSS total score. The model included somatization (SCL-90-R), anxiety, depression, and diagnostic group (dummy-coded: FM-only as reference; IBS-only = 1/FM = 0; FM + IBS = 1/FM = 0) as predictors.

Internal consistency of all psychometric instruments was evaluated using Cronbach’s alpha (Wessa, 2023).

The significance of each coefficient was assessed using *t*-tests, and model fit was evaluated via the adjusted *R*^2^ and *F*-statistic.

Statistical analyses were performed using SigmaStat 11.0 (Systat Software, Inc., San Jose, CA, USA) and GraphPad Prism 5 (GraphPad Software, Inc., La Jolla, CA, USA). A two-tailed *p*-value < 0.05 was considered statistically significant.

Given the exploratory and descriptive nature of this cross-sectional study, no formal *a priori* sample size calculation was performed. The available sample was determined by the number of eligible patients attending the two outpatient clinics during the recruitment period. Nevertheless, the achieved sample size proved sufficient to detect large effect sizes in key outcomes such as somatization and quality of life.

## Results

A total of 53 patients (male/female ratio: 5/48; mean age: 47.43 ± 1.32 years; mean BMI: 25.8 ± 0.8) were enrolled in the study and categorized into three groups: FM (*n* = 13; male/female: 0/13; mean age: 50.69 ± 1.97; mean BMI: 27.69 ± 2.33), IBS (*n* = 18; male/female: 5/13; mean age: 49 ± 2.37; mean BMI: 26.76 ± 1.09), and comorbid FM + IBS (*n* = 22; male/female: 0/22; mean age: 44.18 ± 2.12; mean BMI: 23.88 ± 1.09).

The clinical characteristics of FM patients are reported in Table 1. According to the FIQ-R, patients with FM alone scored within the severe FM category (score range: 48–68), whereas those with comorbid FM + IBS fell into the very severe FM category (score >68). Both groups showed mean FSS scores above the established threshold of 4.67, indicating significant fatigue. Mean pain intensity, assessed using the VAS, was very high in both groups. Evaluation of tender points revealed widespread involvement, with a median count of 18 positive points in each group.

**Table 1:** Multidimensional classification of FM patients.

	FM (Minerbi et al., 2023)	FM + IBS (Enkvist et al., 2023)	*p* value
FIQ-r	64.88 ± 7.11^a^	69.20 ± 5.55^a^	0.79
FSS	5.20 ± 0.37^a^	4.69 ± 0.37^a^	0.55
Tender Point Examination	18 (8–18)^a^	18 (9–18)^a^	0.67
vas pain	78.08 ± 3.82^a^	76.36 ± 2.93^a^	0.91

FM, Fibromyalgia; IBS, Irritable Bowel Syndrome; FIQ-R, Fibromyalgia Impact Questionnaire-Revised; FSS, Fatigue Severity Scale; VAS, Visual Analog Scale. Data are expressed as Mean ± SEM and analyzed by the Mann–Whitney test. Only the tender point number was expressed as Median and range. Differences were considered significant at *p* < 0.05. Values that do not share the same letter are significantly different.

Hematochemical parameters fell within the normal reference ranges across all groups; no further statistical analysis was performed (data not shown).

Regarding the symptom profile assessed with the IBS-SSS questionnaire, significant differences were found among the three groups in both total scores and individual items (Table 2). As expected, the overall IBS-SSS score was significantly higher in the FM + IBS and IBS groups compared to the FM-only group (Kruskal-Wallis test, *p* = 0.0001). This indicates that the severity of IBS-related symptoms is primarily associated with IBS, whether it occurs alone or alongside FM. Consequently, patients in the FM + IBS and IBS groups exhibited moderate symptom severity based on the IBS-SSS classification, while FM-only patients experienced only mild GI symptoms.

**Table 2:** The symptomatic characteristics of FM, IBS and FM + IBS patients.

	FM (Minerbi et al., 2023)	IBS (Drossman and Hasler, 2016)	FM + IBS (Enkvist et al., 2023)	*p* value
IBS-SSS				
Abdominal Pain Intensity	11.15 ± 5.29^a^	42.56 ± 5.65 ^b^	46.82 ± 4.73 ^b^	0.0007
Abdominal Pain Frequency (Days)	6.92 ± 3.28^a^	36.67 ± 7.14^b^	43.64 ± 7.04^b^	0.0004
Abdominal Bloating Severity	28.23 ± 7.64^a^	55.11 ± 4.98^ab^	64.32 ± 5.94^b^	0.003
Dissatisfaction With Bowel Habits	37.31 ± 6.47^a^	62.50 ± 6.13^b^	68.36 ± 5.42^b^	0.004
Symptom Impact on Daily Life	30.62 ± 7.80^a^	56.89 ± 5.59^b^	62.86 ± 4.05^b^	0.004
Total Score	114.2 ± 24.60^a^	253.7 ± 16.83^b^	286.0 ± 17.53^b^	0.0001

FM, Fibromyalgia; IBS, Irritable Bowel Syndrome; IBS-SSS, IBS Symptom Severity Score. Data are expressed as Mean ± SEM and analyzed by the Kruskal–Wallis test with Dunn’s multiple comparison test. Differences were considered significant at *p* < 0.05. Values not sharing the same letter are significantly different.

Except for “abdominal bloating severity,” which showed a statistically significant difference only between the FM and FM + IBS groups but still supported the overall trend, the analysis of individual questionnaire items across all three groups reinforced this pattern. Although the FM group did not meet the Rome criteria for IBS, these patients reported mild GI symptoms, indicating the presence of functional bowel disturbances below the diagnostic threshold for IBS.

All three patient groups showed high stress levels, with no significant differences among them (Table 3). However, the GSI, which indicates overall psychopathological distress based on the SCL-90-R questionnaire, demonstrated a clear progression across the diagnostic categories: from IBS to FM, with further increases in the FM + IBS group (Kruskal-Wallis test, *p* = 0.005). A statistically significant difference was also observed between the IBS and FM + IBS groups.

**Table 3:** Psychological profiles of FM, IBS and FM + IBS patients.

	FM (Minerbi et al., 2023)	IBS (Drossman and Hasler, 2016)	FM + IBS (Enkvist et al., 2023)	*p* value
Stress	19.38 ± 1.27^a^	21.56 ± 1.14^a^	21.36 ± 1.35^a^	0.547
SCL-90-R				
GSI	76.31 ± 5.42 ^ab^	66.72 ± 5.37 ^b^	89.77 ± 4.40 ^a^	0.005
Somatization	83.62 ± 4.50 ^a^	61.06 ± 4.77 ^b^	97.68 ± 4.43 ^a^	<0.0001
Obsessive-Compulsive	69.77 ± 4.05 ^ab^	62.94 ± 3.60 ^b^	81.59 ± 3.62 ^a^	0.003
Interpersonal Sensitivity	59.85 ± 4.41 ^a^	63.67 ± 4.44 ^a^	68.09 ± 5.21 ^a^	0.69 ns
Depression	72.46 ± 5.15 ^ab^	65.72 ± 5.14 ^b^	84.77 ± 4.27 ^a^	0.013
Anxiety	65.46 ± 6.14 ^ab^	60.61 ± 4.79 ^b^	82.18 ± 4.80 ^a^	0.004
Hostility	60.69 ± 5.63 ^ab^	50.72 ± 2.31 ^b^	65.59 ± 4.42 ^a^	0.03

FM, Fibromyalgia; IBS, Irritable Bowel Syndrome; SCL-90-R, Symptom Checklist-90- Revised; GSI, Global Severity Index. Data are expressed as Mean ± SEM and analyzed by the Kruskal–Wallis test with Dunn’s multiple comparison test. Differences were considered significant at *p* < 0.05. Values that do not share the same letter are significantly different.

This pattern was similarly reflected across several subscales of the SCL-90-R, including “Obsessive-Compulsive” (*p* = 0.003), “Depression” (*p* = 0.013), “Anxiety” (*p* = 0.004), and “Hostility” (*p* = 0.03), all showing a progressive increase from IBS to FM to FM + IBS. The “Somatization” scale not only exhibited this sequential trend but also revealed significant differences between IBS and FM (*p* < 0.001) as well as between IBS and FM + IBS (*p* < 0.001). However, no significant difference was observed between FM and FM + IBS.

Furthermore, IBS patients scored below the clinical threshold on the Somatization, Obsessive-Compulsive, Anxiety, and Hostility scales compared to FM + IBS patients, indicating lower levels of psychopathological burden in the former group.

Analysis of health-related QoL using the SF-36 questionnaire revealed meaningful differences among the three groups (Table 4). On the PCS subscale, a significant difference was observed across groups (Kruskal-Wallis test, *p* < 0.001), with FM patients reporting the lowest scores, followed by FM + IBS patients, and IBS patients reporting the highest physical health scores.

**Table 4:** Quality of Life of FM, IBS and FM + IBS patients.

	FM (*n* = 13)	IBS (*n* = 18)	FM + IBS (*n* = 22)	*p* value
SF-36				
PCS	31.69 ± 2.35 ^a^	49.17 ± 3.36 ^b^	38.33 ± 2.49 ^a^	<0.0001
MCS	30.91 ± 4.38 ^ab^	38.14 ± 3.36 ^a^	26.75 ± 2.63 ^b^	0.048
WHOQOL-BREF				
Physical	52.75 ± 2.94^ab^	54.41 ± 3.29^b^	43.56 ± 2.60^a^	0.02
Psychical	55.45 ± 4.18	54.17 ± 2.86	44.70 ± 3.22	0.04
Social	70.52 ± 4.28^a^	62.49 ± 4.69^ab^	50.10 ± 4.72^b^	0.01
Environmental	59.64 ± 3.98^ab^	60.42 ± 3.68^a^	46.99 ± 2.95^b^	0.01

FM, Fibromyalgia; IBS, Irritable Bowel Syndrome; SF-36, 36 Item Short-Form Health Survey; PCS, Physical Component Summary; MCS, Mental Component Summary. Data are expressed as Mean ± SEM and analyzed by the Kruskal–Wallis test with Dunn’s multiple comparison test. Differences were considered significant at *p* < 0.05. Values not sharing the same letter are significantly different.

On the MCS subscale, mental health status gradually declined from IBS to FM to FM + IBS (*p* = 0.048), with a significant difference between the IBS and FM + IBS groups.

According to the WHOQOL-BREF questionnaire (Table 4), patients with FM + IBS reported a lower QoL than those with either condition alone across all four domains. Specifically, the physical domain score was significantly lower in FM + IBS patients compared to those with IBS (*p* = 0.027). No significant differences were found in the psychological domain among the three groups (*p* = 0.04). In the social relationship domain, a significant difference was observed between the FM and FM + IBS groups (*p* = 0.018). Lastly, in the environmental domain, FM + IBS patients scored significantly lower than IBS patients (*p* = 0.017).

The Spearman correlation analysis revealed a significant positive relationship between the IBS-SSS total score and the SCL-90-R “Somatization” subscale (*r* = 0.28, *p* = 0.041), as well as between the IBS-SSS total score and the “Anxiety” subscale (*r* = 0.31, *p* = 0.022). It also identified a significant negative relationship between the IBS-SSS total score and the physical health domain of the WHO-QOL-BREF questionnaire (*r* = −0.29, *p* = 0.037).

Of note, somatization and the diagnostic group independently accounted for half the variance in IBS-SSS (adjusted *R*^2^ = 0.50, *F* = 11.36, *p* < 0.001), with somatization emerging as the most significant independent predictor.

More specifically, both the IBS and FM + IBS groups exhibited a significant increase in IBS-SSS total scores compared to the FM group alone. Among the psychological variables, only the SCL-90-R “Somatization” scale contributed significantly to the severity of IBS symptoms. The “Anxiety” subscale nearly reached statistical significance (Table 5).

**Table 5:** Regression analysis between the IBS-SSS total score and the level of psychological parameters.

Parameters	(β)	Std. Error (*β*)	*p*	95% CI
SCL90-R Anxiety	1.211	0.664	0.074	−0.090–2.512
SCL90-R Depression	- 0.912	0.698	0.198	−2.280–0.456
SCL90-R Somatization	1.303	0.605	0.036	0.114–2.486
IBS group	168.621	29.874	<0.001	110.068–227.174
FM+IBS group	144.407	27.668	<0.001	90.178–198.636

SCL-90-R, Symptom Checklist-90- Revised; IBS, Irritable Bowel Syndrome; FM, Fibromyalgia. A linear regression analysis was performed considering the IBS-SSS as the dependent variable and SCL90 “Somatization”, scale as the independent variables, along with the group IBS and FM+IBS respect to FM alone calculated as dummy variables.

Reliability coefficients ranged from 0.70 to 0.95 across scales, indicating acceptable to excellent internal consistency. Only two SF-36 subscales showed values below this range: Vitality (*α* = 0.68) and Role Emotional (α = 0.63). Although borderline, both coefficients remain within the minimum acceptable threshold for research use.

## Discussion

The concurrent presence of FM and IBS delineates a distinct clinical phenotype characterized by a cumulative burden of somatic, psychological, and functional impairments. Although FM-only and FM + IBS patients reported comparable severity of core FM symptoms, such as pain, fatigue, and tender points, the coexistence of IBS significantly exacerbated GI symptoms, psychological distress, and QoL. Our findings, therefore, suggest that comorbidity should not be viewed merely as the coexistence of two conditions, but rather as an indicator of a more severe and complex phenotype.

A key result of this study is that somatization emerged as the only psychological predictor of IBS symptom severity. This reinforces the notion that GI distress in FM is not solely attributable to peripheral mechanisms but also to psychological processes that amplify symptom perception (Martinez-Martinez et al., 2014). Within the framework of central sensitization, both somatic and visceral hypersensitivity may interact with cognitive-emotional processes, producing heightened awareness of bodily sensations (Veale et al., 1991). Functional neuroimaging studies support this hypothesis, demonstrating increased anterior cingulate cortex activity in response to both somatic and emotional stimuli in patients with FM and IBS (Becker et al., 2011; Rehm et al., 2021).

The progression of psychopathological distress across diagnostic groups, from IBS to FM, and most markedly to FM + IBS, highlights a dose–response relationship between comorbidity and psychological burden. Depression, anxiety, and obsessive-compulsive traits were significantly higher in FM + IBS patients, while IBS-only patients generally scored below clinical thresholds. These results align with earlier evidence that patients with overlapping functional somatic syndromes show poorer psychological outcomes and reduced QoL compared to those with isolated conditions (Rodrigo et al., 2013; Rodrigo et al., 2014).

Our findings also confirm that FM patients, even without a formal IBS diagnosis, report subthreshold GI symptoms. This observation supports increasing evidence of gut-brain axis dysregulation in FM (Settembre et al., 2022). The presence of functional bowel disturbances below diagnostic thresholds suggests that GI involvement may be intrinsic to the FM phenotype, further emphasizing the need for integrated models of care (Erdrich et al., 2020).

From a psychosocial perspective, the significant decline in QoL, especially in the FM + IBS subgroup, extends beyond medical impairment. Reduced QoL (Ucuncu et al., 2020), particularly in FM + IBS, is not only a marker of medical impairment but also a driver of stigma, social withdrawal, and depression. The phenomenon of “invisible illness” reported by FM and IBS patients (Colombo et al., 2025) illustrates how social perceptions can compound symptom burden. These psychosocial dynamics warrant greater attention in both clinical assessment and treatment planning.

Our results endorse a biopsychosocial model of FM and IBS, where symptom severity arises from the interplay of somatic dysregulation, psychological distress, and social factors. This model challenges the traditional biomedical dichotomy between “organic” and “psychological” illness, instead highlighting their mutual reinforcement (Almansa et al., 2009). Routine screening for somatization, stress, and QoL impairments should therefore become standard practice in both gastroenterology and rheumatology clinics.

In terms of intervention, evidence-based psychological approaches such as cognitive-behavioral therapy, or mindfulness-based interventions may help reduce somatization and improve coping with chronic pain and GI distress (Kroenke and Swindle, 2000). By targeting maladaptive cognitive and affective processes, such as catastrophizing, hypervigilance, and emotional dysregulation, these therapies may indirectly alleviate somatic symptoms. Furthermore, integrated care models combining gut-directed therapies (e.g., dietary interventions, neuromodulators) (Becker et al., 2011) with psychological support may be particularly effective in FM + IBS patients (Przekop et al., 2012; Kurland et al., 2006). This phenotypic stratification could guide personalized treatment algorithms, ensuring that high-burden patients receive timely multidisciplinary input.

The present study has some strengths, including the systematic comparison of three well-characterized clinical groups, the use of validated multidimensional instruments, and the focus on the underexplored FM + IBS phenotype.

However, a number of limitations must be acknowledged. First, the cross-sectional design precludes causal inference. Second, the sample size, while sufficient to detect large effect sizes in primary outcomes (e.g., somatization, QoL), was modest and may have limited power to detect smaller differences between groups, particularly in secondary measures such as FIQ-R and FSS. Third, the absence of a healthy control group limits our ability to determine whether observed profiles are specific to these functional disorders or reflect general distress. Fourth, we did not assess small fiber neuropathy (SFN), which has been increasingly implicated in both FM and IBS and may represent a shared peripheral driver of central sensitization. Fifth, a formal *a priori* sample size calculation was not conducted. This decision was justified by the absence of prior data on the expected magnitude of between-group differences in the Italian population, which precluded a reliable estimation of effect size and variance. While this represents a methodological limitation, the large and consistent effect sizes observed across the main outcomes support the robustness and interpretability of our findings. The results should therefore be regarded as hypothesis-generating and valuable for informing future studies with adequate statistical power. Finally, dietary intake was not systematically assessed beyond the exclusion of restrictive regimens, which may have influenced GI symptom reporting.

Future longitudinal, multi-center studies that include biomarker assessments (e.g., inflammatory markers, microbiome) and healthy controls are necessary to confirm these findings and examine underlying mechanisms. Such research should also explore the role of SFN and autonomic dysfunction to better define the biological substrate of this high-burden phenotype.

The present study demonstrates a gradual increase in somatization, psychological distress, and impairment in QoL across the three diagnostic groups, with the FM + IBS group consistently showing the highest burden. This pattern supports the idea that FM and IBS may share some overlapping processes, such as central sensitization, altered interoceptive processing, and gut–brain axis dysregulation, while still having different primary clinical symptoms. However, because of the cross-sectional design, the observed pattern cannot be seen as proof of a causal or mechanistic link. Instead, it suggests a hypothesis-generating model in which FM and IBS are related but not exactly the same expressions of vulnerability to somatic symptom amplification. Therefore, the term “biopsychosocial continuum” should be viewed as a provisional interpretive model rather than a confirmed explanation. Long-term studies, mechanistic research, and biomarker approaches are needed to determine whether the increasing severity from IBS to FM to FM + IBS reflects shared underlying mechanisms, accumulated comorbidities, or separate but parallel clinical courses. Clarifying these mechanisms is crucial to establishing whether the comorbid phenotype is a true additive disorder, a distinct subtype, or the extreme end of a shared symptom spectrum.

## Data Availability

The datasets presented in this study can be found in online repositories. The names of the repository/repositories and accession number(s) can be found at https://doi.org/10.6084/m9.figshare.29436260.v1.
